# LViM: Language-Infused Visual Mamba for apple leaf pests and diseases precise segmentation in complex environments

**DOI:** 10.1016/j.plaphe.2026.100236

**Published:** 2026-06-06

**Authors:** Jiale Chen, Wei Shi, Ziyang Shi, Lin Li, Hao Zhou, Xuanhao Li

**Affiliations:** School of Electronic Information and Physics, Central South University of Forestry and Technology, Changsha, 410004, China

**Keywords:** Crop disease segmentation system, Semantic-visual hybrid network, Multi-scale feature fusion network, Deep learning

## Abstract

Apple leaf disease segmentation is critical for yield and quality preservation in what is globally one of the most economically significant fruit crops. Despite recent advances in deep learning, real-world orchard environments present three primary challenges: (1) low contrast between lesions and background textures, which hinders accurate localization; (2) leaf overlap and occlusion, leading to incomplete feature representation and increased false negatives; and (3) the inherent limitations of unimodal RGB imagery in capturing subtle pathological features, which constrains generalization and accuracy. To address these issues, we proposed Language-Infused Visual Mamba (LViM), a dual-path U-Net architecture that integrates Mamba and Transformer modules for semantic-visual feature fusion. LViM achieves robust segmentation in complex environments through three core innovations: (1) A U-shaped Multimodal Transformer (MTT) branch integrated with AMBERT, which leverages inter-modal semantic relationships to enhance textual feature extraction and provide high-level semantic cues, thereby improving lesion-background discriminability; (2) a U-shaped Visual State Space (VMamba) branch that employs 2D Selective Scanning (SS2D) and Visual State Space (VSS) blocks to capture global context and fine-grained details, mitigating the impact of occlusion; and (3) Cross-Attention Gate Fusion (CAGF) and Linguistic Cross-Nested (LCN) modules that facilitate efficient cross-modal alignment and hierarchical feature modeling to better identify subtle lesions. Experimental results demonstrate that LViM consistently outperforms the VM-UNet baseline, yielding improvements of 4.05% in Precision, 4.25% in Dice coefficient, 4.49% in mIoU, and 4.23% in Recall.

## 1 Introduction

Due to their high nutritional density, apples are a cornerstone of the global agricultural economy and are widely recognized for their health-promoting properties. They serve as a primary subject for the development of intelligent disease identification and control systems, offering critical insights into the multimodal analysis of plant pathology. Rich in vitamin C, polyphenols, boron, and potassium, apples provide various physiological benefits, including significant antioxidant, anticarcinogenic, and cardioprotective effects. From an industrial perspective, apple cultivation generates substantial employment and remains indispensable to the global fruit trade as the most widely cultivated and productive fruit crop. Despite the industry's growth, it faces significant challenges, including substantial yield reductions caused by disease outbreaks and climatic anomalies. Pathogens such as Alternaria leaf spot [1], brown spot [2], gray spot [3], and rust [4] pose severe risks to production, making effective disease management vital for the sustainable development of the industry.

While chemical pesticides remain the primary approach for controlling apple diseases, their prolonged and intensive application has introduced secondary complications. Research indicates that continuous pesticide use can lead to soil and water contamination, which promotes pathogen resistance [5,6]. Furthermore, reliance on chemical intervention increases operational costs and threatens ecological stability and food safety. Consequently, the development of rapid, accurate disease identification and severity assessment technologies is essential for formulating precise, scientifically-grounded control strategies.

Traditionally, early-stage pest and disease identification relied on manual visual inspection, a process that is often inefficient and highly dependent on human expertise. With the proliferation of the agricultural Internet of Things (IoT), image-processing-based detection methods have gained traction. Conventional segmentation techniques typically utilize color space transformations (e.g., HSV, Lab) in conjunction with thresholding or texture features (e.g., GLCM, LBP) to construct classification models. For instance, Wang et al. developed an enhanced K-means clustering algorithm for segmenting crop disease images, providing a highly automated framework [7]. Similarly, Jothiaruna et al. proposed a segmentation method that integrates color characteristics with region growing, achieving an average precision of 87% [8]. However, these traditional approaches often struggle to maintain robust performance when faced with the complexities of real-world agricultural environments.

In 2015, Ronneberger et al. introduced the U-Net architecture, pioneering a paradigm shift in deep learning-based image segmentation [9]. The innovative encoder-decoder structure and skip-connection design of U-Net have significantly advanced the field, making it particularly suitable for specialized domains such as agricultural disease identification. Chen et al. introduced the Atrous Spatial Pyramid Pooling (ASPP) module, which leverages atrous convolutions with varying dilation rates to extract multi-scale features, achieving a Dice coefficient of 0.91 on a leaf disease dataset [10]. Similarly, the SAU-Net model proposed by Wang et al. enhances wheat spike feature extraction by employing Strip Pooling Blocks (SPB) and Multi-Scale Dilated Convolution (MSDC) to capture both local and global context [11]. Despite these successes, U-Net—and convolutional neural networks (CNNs) in general—face three inherent limitations: (1) the localized receptive field restricts the model's ability to model long-range global dependencies, hindering the correlation of lesions across an entire leaf; (2) the inductive bias of translation invariance can lead to suboptimal performance in location-sensitive tasks; and (3) the data-independent nature of convolutional weights prevents the model from dynamically adapting its feature extraction strategy based on the input content.

The introduction of the Vision Transformer (ViT) by Google Research in 2021 addressed several of these CNN-related constraints [12], driving researchers toward more sophisticated attention-based solutions. ViT facilitates global context modeling across image patches through its self-attention mechanism. Furthermore, its innovative positional encoding—utilizing sinusoidal functions or learnable offsets—enables multi-scale disease segmentation by explicitly representing spatial relationships. For instance, Alwan et al. proposed TransPlant, which integrates the Pyramid Vision Transformer (PVT) with a multi-scale feature fusion module, achieving a mIoU of 87.6% on grape black spot and downy mildew segmentation—a 9.3% improvement over U-Net, with notably better performance along lesion boundaries [13]. Additionally, Ishibashi et al. introduced a token pruning method incorporating Variable Proportion Attention (VPA) and Gradient-Aware Scaling (GAS) [14]. On the CIFAR-10 dataset, this approach achieved a precision of 80.62% while reducing the error rate by 50.44% and latency by 46.8%, demonstrating an effective trade-off between accuracy and computational efficiency.

However, despite the advantages of Vision Transformers over CNNs, two primary challenges remain. First, the self-attention mechanism entails a computational complexity that scales quadratically with the input resolution, leading to prohibitive memory demands for high-resolution imagery. Second, ViT models are highly susceptible to overfitting when trained on limited datasets, which constrains their utility in domains where annotated data is scarce and hinders their deployment in practical agricultural scenarios.

Developed by a research team at the Chinese Academy of Sciences in 2024, VMamba has emerged as a pivotal architecture for crop segmentation [15]. It effectively integrates the linear time complexity and global context modeling capabilities of Visual State Space (VSS) blocks with the 2D Selective Scanning (SS2D) mechanism. In 2025, Zhang et al. introduced DD-HTL VMamba, a method combining VMamba with diffusion models and transfer learning. Evaluated on the PlantVillage dataset, this approach demonstrated a 3.49% precision improvement over the Swin Transformer while reducing training time by 80% [16]. Furthermore, Ruan et al. developed VM-UNet, achieving a Dice Similarity Coefficient (DSC) of 89.03% in medical imaging, outperforming various mainstream benchmarks [17]. Similarly, Shi et al. proposed LUNet for *Camellia oleifera* pest and disease segmentation, yielding an mIoU of 91.92%—a 5.12% increase over the TransUNet baseline [18].

Despite significant advancements in segmentation methodologies, three primary challenges persist: (1) dense canopy conditions result in minimal textural contrast between diseased regions and healthy backgrounds, hindering precise lesion localization; (2) leaf overlap and occlusion obscure pest-damaged areas, leading to fragmented feature representations; and (3) a heavy reliance on unimodal RGB imagery precludes the capture of subtle pathological symptoms, limiting adaptability across diverse field conditions. To address these gaps, it is essential to develop more resilient algorithmic architectures that enhance robustness in complex agricultural environments without compromising segmentation precision.

Conventional unimodal segmentation models typically rely on a standalone encoder-decoder architecture for feature extraction and compression. However, such frameworks are highly susceptible to environmental noise, which often compromises the feature extraction process [19]. Unimodal features frequently lack the necessary representational capacity, failing to encapsulate both fine-grained details and high-level semantic information—both of which are critical for accurate detection [20]. Consequently, these models often exhibit suboptimal performance in unstructured open environments, where variable illumination and background clutter (e.g., weeds) impede stable segmentation. Standard unimodal detection technologies thus struggle to meet the rigorous demands for precision required in apple leaf disease analysis.

The identification of lesions in complex agricultural environments has long been a significant challenge. However, the integration of deep learning with multimodal data provides a viable pathway for enhancing disease segmentation accuracy by leveraging complementary features. While unimodal learning often fails to fully capture the diverse characteristics of apple leaf diseases, multimodal approaches enable a multi-dimensional representation of pathological features. Significant progress has been made in semantic segmentation through the alignment of textual and visual information. Environmental data and disease-related descriptions often contain latent patterns regarding disease occurrence; by implicitly incorporating these cues into model training, we can enhance segmentation precision and gain a more comprehensive understanding of pest and disease manifestations. This, in turn, facilitates the optimization of disease control measures and mitigates economic losses.

**Fig. 1:**
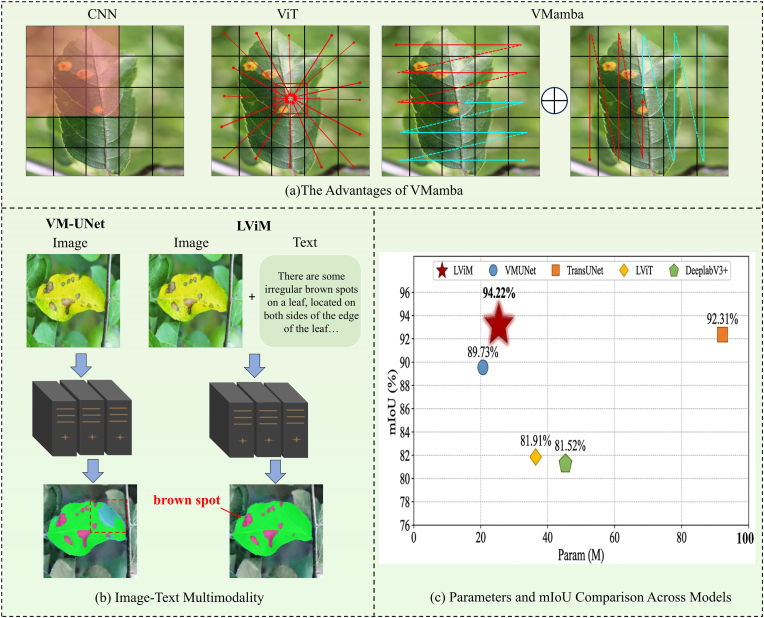
(a) Comparison of the 2D Selective Scanning (SS2D) mechanism in VMamba versus spatial domain traversal in CNN and ViT models. (b) Architectural distinction between the proposed multimodal LViM model, featuring semantic-visual hybrid encoding, and the unimodal VM-UNet baseline. (c) Comparison of parameters and mIoU values between the proposed LViM and other models shows that LViM outperforms Transformer-based models in terms of mIoU, while maintaining only 26% of the parameters of TransUNet.

In this study, we propose Language-Infused Visual Mamba (LViM), a Mamba-Transformer dual-path U-Net architecture designed for semantic-visual feature fusion in apple leaf disease segmentation. Building upon the VM-UNet framework, LViM consists of two primary components: a U-shaped Visual State Space (VMamba) path for visual feature processing and a U-shaped Multimodal Transformer (MTT) branch [21] for cross-modal feature fusion. Leveraging the lightweight architecture of VMamba, the overall model achieves a substantial reduction in parameter count while preserving robust representational capacity (Fig. 1(c)). The MTT module is specifically engineered to interface with AMBERT [22] to extract discriminative textual features through multimodal semantic correlations while suppressing redundant noise. To ensure architectural compatibility between visual and linguistic modalities, a Cross-Attention Gate Fusion (CAGF) module [23] is strategically integrated into the VMamba skip connections. This design enables the effective modeling of cross-modal correlations while preserving critical local spatial details. Furthermore, we implement a Linguistic Cross-Nested (LCN) module [24] to further refine segmentation performance through enhanced semantic understanding and the collaborative integration of linguistic and image data.

The primary contributions of this study are summarized as follows:

1) We leverage VMamba as the core visual encoder for apple leaf disease segmentation. By utilizing the Visual State Space (VSS) block and the 2D Selective Scanning (SS2D) mechanism, this architecture employs a four-way cross-scanning strategy to effectively capture global context information (Fig. 1(a)). This design achieves high-precision segmentation of lesion areas while maintaining linear time complexity, significantly improving performance over traditional CNN and ViT-based models.
2) We introduce the Cross-Attention Gate Fusion (CAGF) module. By dynamically modulating attention weights across both channel and spatial dimensions, this module enhances the network's ability to extract fine-grained lesion details and precise target localization. Furthermore, it effectively integrates the global semantics captured by VMamba with local spatial features, ensuring boundary integrity and improving segmentation accuracy in complex orchard environments.
3) We proposed an innovative integration of the AMBERT text encoder, the Multimodal Transformer (MTT) module, and the Linguistic Cross-Nested (LCN) module to enhance cross-modal feature fusion through their synergistic effects (Fig. 1(b)). Specifically, AMBERT processes fine- and coarse-grained token sequences simultaneously to generate multi-scale textual representations. The MTT module facilitates efficient interaction between linguistic and visual data, while the LCN module further deepens this collaboration by constructing a semantic-guided visual encoding framework that realizes deep, cross-scale integration of text embeddings and image features.
4) In collaboration with plant pathology experts, we have systematically constructed a comprehensive multimodal dataset for apple disease analysis. This dataset comprises image-text pairs captured in natural environments, covering four major disease types: Alternaria leaf spot, brown spot, gray spot, and rust. This resource provides a foundation for advanced multimodal research in agricultural plant protection.

## 2 Related work

This section delineates the three foundational technologies underpinning the proposed LViM framework: text-image feature fusion, attention mechanisms, and the dual-path U-Net architecture. We first discuss the significance of integrating textual and visual data for crop image segmentation, followed by a review of existing technical solutions and a detailed explanation of our proposed multimodal fusion module and its functional advantages. Furthermore, we explore the critical role of these modules in processing multimodal datasets and introduce the Cross-Attention Gate Fusion (CAGF) module within the context of attention-based learning. Finally, we discuss the evolution of dual-path U-Net architectures in agricultural contexts, clarifying the design philosophy and performance benefits of the Mamba-Transformer dual-path U-Net that constitutes the core of this research.

### 2.1 Text-image feature fusion methods

Multimodal feature fusion is a fundamental area of research focused on integrating the complementary representative strengths of diverse data sources. Different modalities—including images, text, and sensory data—exhibit unique representational characteristics. Rudimentary fusion techniques, such as element-wise addition [25], direct concatenation of heterogeneous features [26], or Hadamard products [27], often introduce redundant information and may even impede effective feature propagation. In contrast, principled integration strategies can significantly enhance feature representation, thereby increasing both discriminability and informational richness. Such strategies are widely employed across various computer vision tasks, including semantic segmentation and image captioning. While early research focused on basic fusion operations due to their computational simplicity, these methods often lacked theoretical depth and optimization. As the field evolved, more complex paradigms emerged, with current state-of-the-art methods categorized into decision-level, feature-level, hybrid-level, and model-level integration. However, these approaches remain susceptible to performance degradation caused by cross-modal semantic misalignment and feature redundancy. To mitigate these issues, this study adopts the MTT module, specifically tailored for the AMBERT language model. The MTT module excels at extracting discriminative textual features while suppressing noise interference, thereby maximizing the utility of cross-modal semantics. Concurrently, we introduce the LCN module to enhance the synergy between linguistic and visual data through cross-scale fusion, effectively filtering redundant information to highlight core pathological features.

### 2.2 Attention module

The attention module has emerged as a fundamental building block in deep learning, enabling adaptive feature selection and dynamic weight redistribution. Inspired by the selective cognitive patterns of the human visual system, this mechanism allows models to focus on the most salient regions of an input. Bahdanau et al. first systematically implemented attention mechanisms within neural machine translation tasks [28], a paradigm that Woo et al. subsequently extended to computer vision for object detection and image classification [29]. Since then, the Transformer architecture has established itself as a cornerstone of the field by adopting an attention-only framework. Today, standard attention and self-attention mechanisms are ubiquitous in both visual and linguistic tasks, having evolved into numerous specialized variants to address diverse computational challenges [30].

However, contemporary multi-scale feature learning methods frequently suffer from scale-specific inductive bias. By prioritizing feature extraction at a particular scale, these methods often overlook complementary information across disparate scales. In the context of crop disease segmentation, for instance, existing frameworks may overemphasize the global semantic context of lesions while neglecting their relationship with the leaf background; conversely, they may focus excessively on localized details while failing to capture subtle edge textures. To address these limitations, we propose the CAGF module, which effectively integrates multi-scale feature representations while preserving fine-grained local details within lesion regions. By synergizing advantages across different scales, the CAGF module mitigates the constraints of traditional attention mechanisms, ensuring more robust and precise segmentation.

### 2.3 Dual-path U-Net architecture

The dual-path U-Net [31] represents a significant evolution of the conventional single-path architecture, designed to overcome the inherent limitations of feature extraction within a single-backbone network. By exploiting the functional complementarity of parallel paths, this architecture circumvents the performance bottlenecks imposed by its single-stream predecessors. Over time, dual-path configurations have matured from rudimentary dual-CNN branches to sophisticated hybrid frameworks that integrate diverse feature extraction paradigms, such as State Space Models (SSMs) and Transformers. Today, this architecture is a cornerstone for intensive prediction tasks, including agricultural disease segmentation and medical image analysis. Compared to the standard U-Net, the dual-path approach captures multi-dimensional feature information through collaborative modeling. This not only mitigates the tendency of single-path models to overlook either local details or global context but also enhances feature discriminability through cross-path interaction, thereby significantly improving segmentation stability and accuracy in complex environments.

Various branch combinations have been explored within the dual-path U-Net framework. For instance, CNN-CNN dual-branch architectures [32] extract fine textures and coarse-scale semantics using distinct kernels and sampling strategies. CNN-Transformer dual-path models [33] aim to harmonize the global modeling capabilities of Transformers with the inductive biases of CNNs. However, these combinations exhibit notable constraints. CNN-CNN frameworks struggle to capture long-range dependencies, making them less effective for modeling cross-regional lesions in agricultural settings. While CNN-Transformer architectures achieve global-local integration, the quadratic computational complexity of the self-attention mechanism results in a prohibitive overhead when processing high-resolution leaf imagery. Furthermore, the significant disparity in feature representation spaces between CNNs and Transformers often leads to misalignment during cross-branch feature fusion. In addition, existing CNN-Transformer dual-path U-Nets generally rely on CNN branches to extract local texture features and Transformer branches to model global dependencies. Their fusion process is therefore mainly based on visual feature complementarity, while explicit semantic guidance from linguistic information is rarely considered.

To address these limitations, this paper proposes a Mamba-Transformer dual-path U-Net architecture. Different from existing CNN-Transformer dual-path U-Nets, the proposed architecture replaces the conventional CNN branch with a VMamba branch. By introducing Visual State Space blocks and the 2D Selective Scanning mechanism, the VMamba branch can capture long-range spatial dependencies with linear computational complexity, making it more suitable for high-resolution apple leaf disease segmentation. Meanwhile, the Transformer branch in LViM is not merely used for visual global modeling, but is coupled with AMBERT-based textual features to provide disease-related semantic priors. To effectively capture global context with linear complexity while preserving fine-grained local features—such as lesion edges and textures—one branch utilizes the VMamba module. Simultaneously, the other branch employs a Transformer to enhance semantic association modeling within lesion regions through its cross-scale attention mechanism. This dual-path configuration overcomes the computational inefficiency associated with high-resolution CNN-Transformer models and the long-range modeling deficiencies of CNN-CNN architectures. Moreover, the complementary nature of VMamba's state-space representations and Transformer's attention-based features effectively alleviates the issue of feature misalignment. Thus, the structural innovation of LViM lies in replacing CNN-based local visual extraction with VMamba-based linear global visual modeling, while further integrating Transformer-based cross-modal semantic fusion, rather than simply combining CNN and Transformer visual features. This integrated approach harmonizes global distribution information with precise local detail extraction, providing an optimized solution for disease segmentation in challenging agricultural scenarios.

## 3 Materials and methods

### 3.1 Image acquisition and processing

**Fig. 2:**
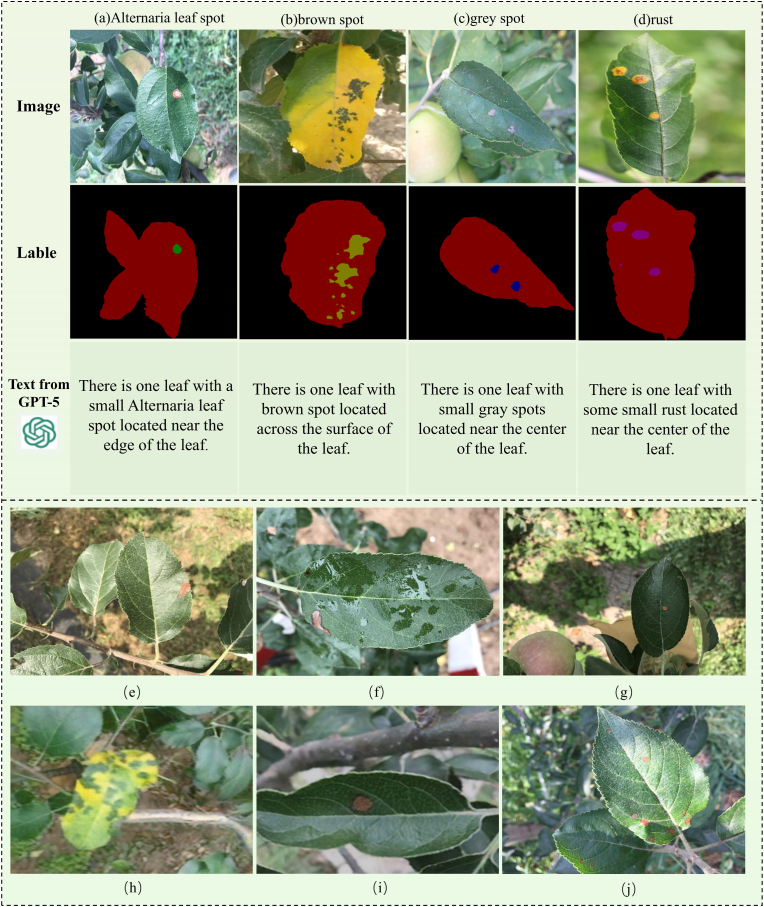
Overview of the multimodal apple leaf disease dataset and annotation framework. (a–d) Typical symptoms and corresponding pixel-level annotations for: (a) Alternaria leaf spot, (b) brown spot, (c) gray spot, and (d) rust, accompanied by GPT-5-generated linguistic descriptions. (e–j) Representative samples of diverse environmental conditions included in the dataset: (e) raindrops, (f) intense illumination, (g) shadows, (h) soil background, (i) stems, and (j) weed interference.

The images used in this study were collected from multiple publicly available sources, including the PlantVillage dataset and publicly accessible web image resources. After manual screening, duplicate removal, category reorganization, and expert annotation, we constructed a multimodal apple leaf disease dataset comprising four representative disease categories, as illustrated in Fig. 2: (a) Alternaria leaf spot, characterized by circular or irregular dark brown to black necrotic lesions, often encircled by a chlorotic halo or distinct concentric rings [34]; (b) brown spot, identified by large, irregular yellowish-brown lesions with dark greenish-brown margins that eventually coalesce into grayish-white centers with dense black acervuli [35]; (c) gray spot, manifesting as near-circular lesions with light brown to grayish-white centers and slightly elevated purple-brown borders, occasionally leading to central cracking or perforation in advanced stages [36]; and (d) rust, initially appearing as yellow-green specks on the adaxial leaf surface that develop into bright orange spots, with characteristic hair-like aecial protrusions on the abaxial surface ranging from yellowish-orange to reddish-brown [37].

A total of 1800 high-quality images were manually curated from the primary dataset, with 80% (1440 images) allocated for training and 20% (360 images) reserved for testing. The dataset covers four representative apple leaf diseases, including Alternaria leaf spot, brown spot, gray spot, and rust. Specifically, it contains 500 images of Alternaria leaf spot, 300 images of brown spot, 500 images of gray spot, and 500 images of rust. Although the brown spot category contains fewer samples than the other categories, data augmentation strategies, including adaptive brightness adjustment, random horizontal flipping, and random rotation, were applied during training to mitigate the influence of class imbalance and improve model robustness. The annotation process followed a collaborative expert-led mechanism. Initial pixel-level segmentation was performed using LabelMe v5.3.1, where the leaf blade was masked in red, while Alternaria leaf spot (a), brown spot (b), gray spot (c), and rust (d) were color-coded in green, yellow, blue, and purple, respectively (see Fig. 2). To facilitate multimodal feature alignment and enhance model performance, the GPT-5 large language model was leveraged to generate discriminative linguistic descriptions for each disease category. It should be noted that GPT-5 was used only to generate category-level disease descriptions as auxiliary textual prompts. These prompts summarize typical pathological characteristics, such as lesion color, morphology, and texture, and serve as semantic priors for multimodal feature learning. GPT-5 is not used as a trainable component of the proposed LViM model and does not participate in the inference stage to generate disease predictions or segmentation masks. Therefore, the final segmentation results are produced by the proposed semantic-visual fusion network rather than directly leveraging the diagnostic capability of GPT-5. The generated textual prompts direct the model to focus on specific pathological features and improve localization accuracy. Furthermore, the dataset incorporates diverse environmental variables—including intense illumination (e), raindrops (f), shadows (g), soil background (h), stems (i), and weeds (j)—to ensure the robustness and generalization of the trained model in real-world orchard conditions.

### 3.2 Deep learning method

#### 3.2.1 Overall architecture

To address critical challenges in apple leaf disease segmentation—such as cross-modal feature misalignment, the loss of fine-grained local details, and diminished feature discriminability in complex backgrounds—this study proposes the Language-Infused Visual Mamba (LViM). LViM is a dual-path U-Net framework designed for synergistic semantic-visual feature fusion to facilitate precise pathological segmentation. The core architecture comprises complementary dual branches based on VMamba and a Multimodal Transformer (MTT). As the visual encoder, VMamba leverages Visual State Space (VSS) blocks and the 2D Selective Scanning (SS2D) mechanism to effectively capture global context while maintaining linear computational complexity. Concurrently, a Cross-Attention Gate Fusion (CAGF) module is integrated into the skip connections of the VMamba path to enhance the extraction of localized lesion details and improve target localization. To establish a robust cross-modal collaborative system, we further incorporate the AMBERT text encoder, the MTT module, and the Linguistic Cross-Nested (LCN) module. This integration constructs a semantic bridge between textual and visual modalities, enabling deep cross-scale fusion and precise alignment of multimodal features. By mitigating the feature interference and semantic ambiguity inherent in unimodal encoding and conventional fusion strategies, LViM significantly improves segmentation accuracy, boundary integrity, and model generalization in complex agricultural environments.

#### 3.2.2 U-VMamba branch

**Fig. 3:**
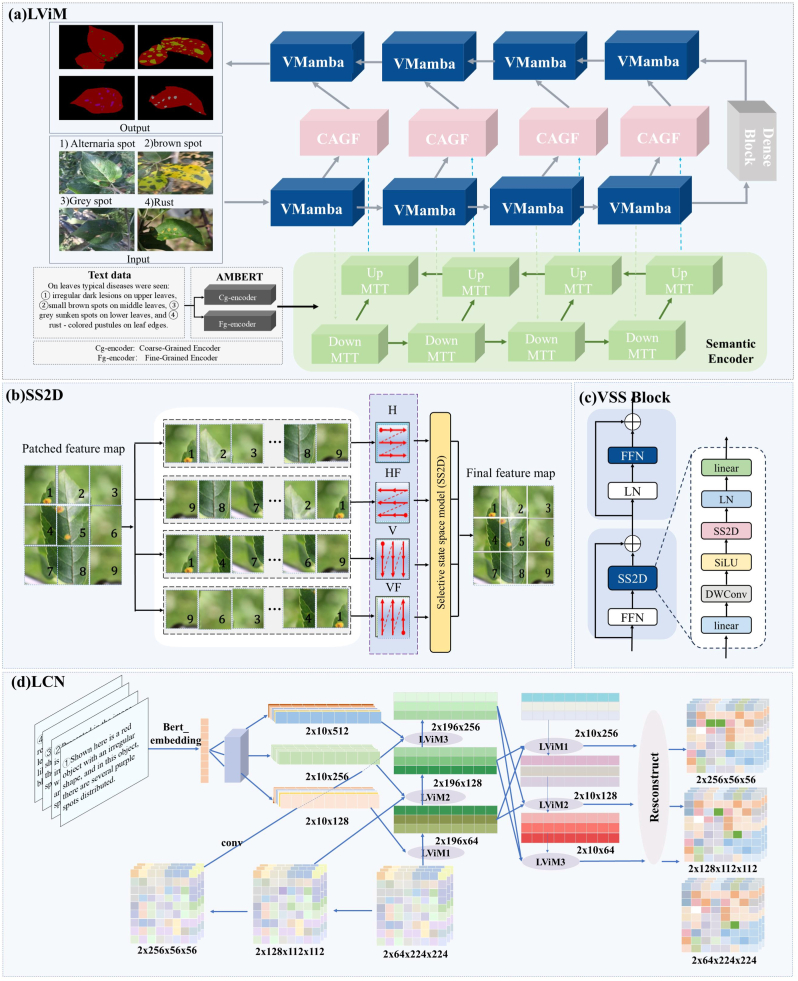
Structural components and innovations of the LViM framework: (a) Overall operational workflow of LViM; (b) 2D-Selective-Scan (SS2D) illustration; (c) Internal structure of the VSS block; (d) Operational flow of the Linguistic Cross-Nested (LCN) module.

As illustrated in Fig. 3(a), the U-shaped VMamba branch processes image inputs to generate the final segmentation masks. VMamba is a visual backbone based on the State Space Model (SSM), designed to learn hierarchical visual representations by optimizing performance, architectural scaling, and visual adaptation. The theoretical foundation of VMamba stems from the Mamba architecture in Natural Language Processing (NLP); its primary innovation lies in extending the selective SSM mechanism—originally designed for 1D sequences—to 2D visual data. The continuous-time evolution of this system is defined by the following differential equations:

(1) $$\begin{array}{c} \;\left\{ \begin{array}{l} h^{\prime }\left(t\right)=Ah\left(t\right)+Bu\left(t\right)\\ y\left(t\right)=Ch\left(t\right)+Du\left(t\right) \end{array}\right.\; \end{array}$$

Where (A,B,C,D) represent the learned weight parameters. To integrate this system into a deep learning framework, the continuous SSM must be discretized. Using a time-scale parameter Δ, the continuous parameters are transformed into their discrete counterparts $\left(\bar{A},\bar{B}\right)$ typically via the zero-order hold (ZOH) method. The resulting discretized state transition is expressed as:

(2) $$\begin{array}{c} h_{b}=\exp\left(A\left(\Delta_{a}+\ldots +\Delta_{b-1}\right)\right)\left(h_{a}+\sum_{i=a}^{b-1}B_{i}u_{i}\;\exp\left(-A\left(\Delta_{a}+\ldots +\Delta_{i}\right)\right)\Delta_{i}\right) \end{array}$$

This discretization enables the model to perform parallelizable computations during training while maintaining linear computational complexity, effectively bridging the gap between continuous signal modeling and efficient sequence learning.

While the selective scanning mechanism of the original Mamba is optimized for 1D sequences [38], visual data possesses an inherent 2D spatial structure that requires specialized traversal. To bridge the gap between 1D scanning and 2D spatial modeling, VMamba introduces the 2D-Selective-Scan (SS2D) module (Fig. 3(b)). Initially, the input image $\begin{array}{c} I\in {\;R}^{H\times W\times 3} \end{array}$ is partitioned into patches by a stem layer, generating a feature map with spatial dimensions of H/4×W/4. This approach preserves the spatial hierarchy of the image without necessitating additional positional encodings. The network comprises four hierarchical stages; excluding the first stage, each subsequent phase begins with a downsampling layer that reduces the feature map dimensions to H/8×W/8, H/16×W/16, and H/32×W/32, respectively, facilitating a multi-scale “fine-to-coarse” feature extraction.

At the core of each stage is the Vision-adapted State Space (VSS) block (Fig. 3(c)). In this architecture, the standard S6 module found in Mamba is replaced by the SS2D module. To further enhance computational efficiency, the redundant multiplication branch from the original Mamba block is removed, resulting in a streamlined single-path network complemented by two residual modules. This design mirrors the structural efficiency of conventional Transformer blocks, achieving an optimal trade-off between representational capacity and computational throughput.

The SS2D module serves as the foundational component of the VSS block, facilitating effective 2D spatial modeling through a tripartite process: cross-scanning, selective scanning, and cross-merging. In the initial stage, a cross-scan operation expands the 2D feature map into four complementary 1D sequences along distinct traversal paths (e.g., horizontal, vertical, and their respective reverse directions). This strategy ensures that each pixel or patch integrates global context through multi-directional traversal. In the second stage, selective scanning employs independent S6 blocks to process these sequences in parallel. These S6 blocks maintain linear complexity by leveraging an input-dependent selection mechanism, where dynamic weights are derived from the discretized SSM parameters to capture complex spatial dependencies. Finally, the cross-merge stage integrates the multi-directional spatial information and reshapes the output of the four S6 blocks back into a 2D feature map. The operational logic of SS2D can be functionally mapped to the following attention-like representation:

(3) $$\begin{array}{c} Y^{\left(j\right)}=\left(Q⊙w^{\left(j\right)}\right)h_{a}^{\left(j\right)}+\left[\left(Q⊙w^{\left(j\right)}\right){\left(\frac{K}{w^{\left(j\right)}}\right)}^{⊤}⊙M\right]V^{\left(j\right)} \end{array}$$

where the discretized SSM feature mappings are represented by (Q,K,V), w denotes the cumulative weights along the scanning path, and M represents the lower-triangular temporal mask. This structure preserves the dynamic weighting capability inherent in self-attention while reducing computational complexity from $O\left(N^{2}\right)$ to O(N).

This mechanism underpins the primary advantages of VMamba. First, its linear scalability ensures high computational efficiency for high-resolution downstream tasks, such as semantic segmentation, as memory consumption and FLOPs scale linearly with input resolution. Second, SS2D establishes a globally unified Effective Receptive Field (ERF) that covers the entire image uniformly. Due to its four-way quadrant scanning architecture, it significantly outperforms comparable SSM-based models like Vim by more accurately capturing local textures and long-range spatial correlations. These attributes specifically address the core challenges in apple leaf disease segmentation, namely the inadequate integration of fine-grained details with global features and the prohibitive cost of processing high-resolution inputs. Consequently, the proposed model provides a superior visual representation solution, offering substantial gains in segmentation precision, processing throughput, and practical field applicability.

#### 3.2.3 U-MTT module

As illustrated in Fig. 3(a), the U-shaped Multimodal Transformer (MTT) module is the central component of the U-MTT branch, specifically engineered to fuse linguistic and visual representations. The fusion process employs a hierarchical and progressive strategy to facilitate robust cross-modal interaction, thereby strengthening the semantic correlations between modalities. Visual and textual data streams are initially processed independently within the MTT framework. The pre-trained AMBERT model serves as the language backbone, performing dual-stream encoding across both fine-grained (Fg-encoder) and coarse-grained (Cg-encoder) textual inputs. Simultaneously, the initial VMamba downsampling layer extracts preliminary visual features from the input image. These processes are formally defined as follows:

(4) $$\begin{array}{c} {\;x}_{text}=Y_{AMBERT}\left({input}_{text}\right)\; \end{array}$$

(5) $$\begin{array}{c} {\;x}_{img}=Y_{DownCNN,1}\left({input}_{img}\right) \end{array}$$

(6) $$\begin{array}{c} Y_{DownMTT}=MTT\left(x_{img},x_{text}\right)\; \end{array}$$

Where ${input}_{img}$ and ${input}_{text}$ represent the input image and textual data streams, respectively; $x_{img}$ denotes the features extracted by the initial VMamba downsampling layer, and $x_{text}$ signifies the textual features encoded by AMBERT. The resulting fused features generated by the MTT module are represented as $Y_{DownMTT}$.

Architecturally, each MTT module follows a standard Transformer encoder configuration, comprising multi-head self-attention (MHSA) mechanisms, multi-layer perceptrons (MLP), and layer normalization, augmented with convolutional operations and non-linear activation functions. In subsequent stages, each downsampling MTT layer integrates the hierarchical features from the preceding MTT module with the corresponding feature maps from the parallel VMamba path. This interaction is expressed as:

(7) $$\begin{array}{c} Y_{DownATT,i+1}=MTT\left(Y_{DownMTT,i}+x_{img,i+1}\right) \end{array}$$

The outputs of the upsampling MTT module are subsequently directed to the CAGF components. Prior to processing within the upsampling VMamba module, these features are integrated with the corresponding downsampling VMamba features—typically through concatenation or summation—to ensure high-level semantic alignment across the dual paths.

**Fig. 4:**
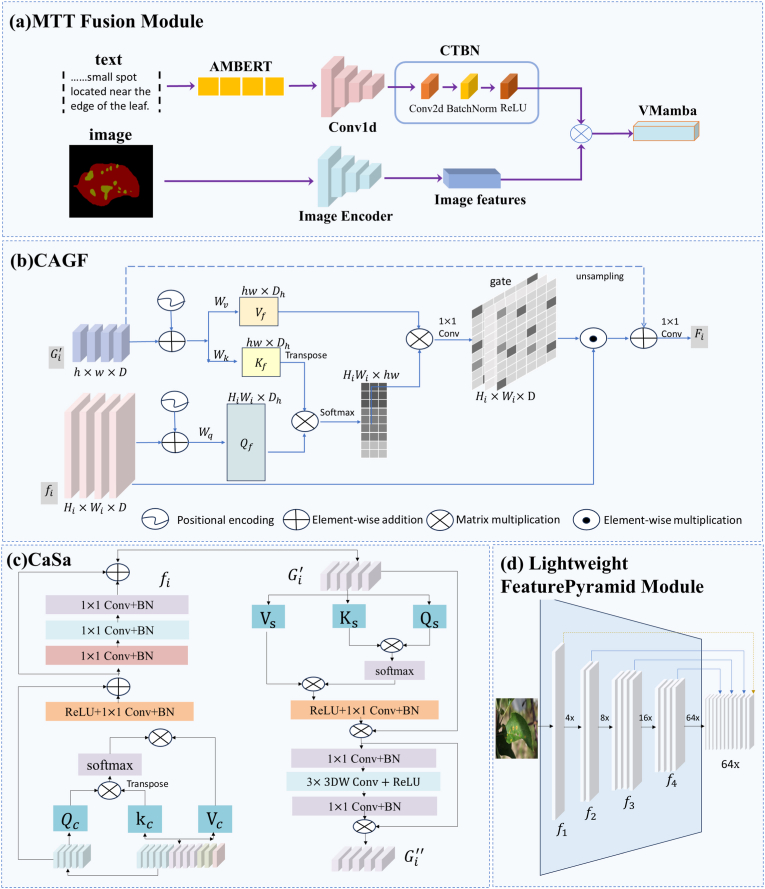
(a) Fusion architecture of the Multimodal Transformer (MTT) module. (b) Schematic of the Cross-Attention Gate Fusion (CAGF) module. (c) Internal structure of the CaSa block for global feature extraction. (d) Lightweight feature pyramid module designed for local feature representation.

To enhance the efficacy of AMBERT in extracting textual features and to model the complex semantic correlations between linguistic and visual modalities, we propose an AMBERT-aligned MTT module, as illustrated in Fig. 4(a). This module is specifically engineered to refine multimodal fusion accuracy through a structured processing pipeline. Initially, the AMBERT backbone ($Y_{AMBERT}$) generates dual-path textual representations encompassing both coarse- and fine-grained semantic information. These features are subsequently passed through a Convolutional Transformer BERT Network (CTBN) layer, which is tailored for the AMBERT output. The CTBN layer consists of two 2D convolutional layers (Conv2d), each followed by Batch Normalization (BN) and Rectified Linear Unit (ReLU) activation, to transform the textual embeddings into a compatible feature space. Cross-modal fusion is then achieved via element-wise multiplication with the visual features ($X_{img}$). Finally, a VMamba block processes the integrated representations to produce the fused feature map (${\;Y}_{MTT}$). The overall transformation is formulated as follows:

(8) $$Y_{MTT}=Y_{VMamba}\left[X_{img}⊗Y_{CTBN}\left(Y_{AMBERT}\left(X_{text}\right)\right)\right]$$

where $X_{img\;}$ and $X_{text}$ denote the image and text inputs, respectively, while $Y_{CTBN}$ and $Y_{VMamba}$ represent the transformation functions of the CTBN layer and the VMamba module. Compared to conventional fusion techniques, the AMBERT-aligned MTT module effectively captures deep cross-modal semantic relationships and generates more discriminative textual representations. As a modular enhancement to the hierarchical U-Net architecture, this design demonstrates superior performance in lesion feature recognition and segmentation accuracy, particularly within the challenging environmental conditions of real-world orchards.

#### 3.2.4 Cross-attention gating fusion (CAGF) module

Specifically designed to bridge the representational divide between local fine-grained visual features and global semantic features, the CAGF module (Fig. 4(b)) serves as a critical complementary unit in the multimodal feature fusion architecture of U-VMamba and U-MTT. An attention gating mechanism is employed to achieve precise complementarity and efficient fusion of the two feature categories. This mechanism effectively filters feature noise and enhances salient semantic representations. This further enhances the segmentation accuracy and feature discrimination capability of the overall model for diseased regions on apple leaves by providing refined support for the hierarchical feature extraction of U-VMamba and the cross-modal fusion of U-MTT. U-VMamba and U-MTT's hierarchical feature processing logic is profoundly compatible with the feature inputs of the CAGF module. In particular, the global features are the fused U-MTT multimodal global features $\begin{array}{c} {G_{i}}^{\prime \prime }\in R^{h\times w\times D},\left(h=H/64,w=W/64,i=2,3,4\right)\; \end{array}$ that are output by the CaSa module (Fig. 4(c)), while the local features are the U-VMamba local features that are extracted by the lightweight feature pyramid module (Fig. 4(d)). Local and global features are condensed into sequential features along the spatial dimension to accommodate the attention mechanism's sequential feature processing. These features are denoted as $\begin{array}{c} f_{i}\in R^{H_{i}\times W_{i}\times D}\;\text{and}\;{G_{i}}^{\prime \prime }\in R^{h\times w\times D} \end{array}$, respectively. Given the spatial position sensitivity of leaf disease features, which are highly correlated with the global leaf structure in terms of the edges, textures, and distribution locations of lesions, and the fact that both U-VMamba and U-MTT hierarchical features contain explicit spatial position information, sine positional encoding (PE) is introduced for the two types of sequential features. While capturing the relative positional relationships between features, a unique code is generated for each spatial position by leveraging the periodicity and boundedness of sine and cosine functions. The encoding procedure is illustrated in Equations (9) and (10):

(9) $$PE\left(pos,2i\right)=\sin\left(\frac{pos}{10000^{\frac{2i}{D}}}\right)$$

(10) $$\begin{array}{c} PE\left(pos,2i+1\right)=\cos\left(\frac{pos}{10000^{\frac{2i}{D}}}\right) \end{array}$$

In the encoding scheme, pos denotes the sequence position, where pos∈[1,hw] for global features and $\begin{array}{c} pos\in \left[1,hw\right] \end{array}$ for local features. The even and odd indices of the feature dimension are represented by 2i and 2i+1, respectively. Specific periodic values from sinusoidal functions are assigned to various spatial positions within the same channel, while the frequencies are varied across dimensions based on the channel index. This ensures the uniqueness of the spatial embeddings and facilitates the capture of relative positional correlations. Consequently, this encoding strategy generates complementary features that align with the cross-modal spatial matching of U-MTT and the spatial perception of U-VMamba. More importantly, spatial positional encoding enables the attention mechanism to retain the coordinate information of lesion pixels during global-local feature interaction. In complex orchard backgrounds, lesion boundaries are easily disturbed by leaf veins, shadows, overlapping leaves, and background textures. Without positional constraints, the attention map may incorrectly respond to visually similar but spatially irrelevant regions. By embedding spatial location information into local and global features, the CAGF module can establish more accurate correspondences between semantic cues and lesion boundary positions, thereby reducing attention drift, preserving edge continuity, and improving the precise localization of lesion boundaries in complex backgrounds.

The CAGF module implements a gated fusion logic based on the cross-attention mechanism, which is architecturally consistent with the Transformer framework in the U-MTT branch. The query features $Q_{f}\in R^{H_{i}W_{i}\times D_{h}}$ are obtained via linear projection of the position-encoded local features. Similarly, the key features $K_{f}\in R^{hw\times D_{h}}$ and value features $V_{f}\in R^{hw\times D_{h}}$ are derived from the global features, where $D_{h}$ signifies the hidden dimension of the mapping. A correlation attention map is computed through the scaled dot-product of $\begin{array}{c} Q_{f}K_{f}^{T} \end{array}$ across all spatial coordinates. This map captures the semantic guidance provided by global attributes (e.g., disease category and macroscopic distribution) over local visual cues (e.g., punctiform lesions and edge contours). By multiplying the attention map with $\begin{array}{c} V_{f} \end{array}$, the module generates semantic significance weights for each local spatial position. These weights function as an attention gate, effectively suppressing irrelevant noise—such as cluttered backgrounds and variable illumination—while highlighting salient regions of interest, such as lesion areas. The localized representations are further fortified by an element-wise multiplication of the cross-attention gate with the original local features, followed by channel-wise addition with upsampled global features to achieve a comprehensive integration of spatial details and semantic context.

Finally, a 1×1 convolutional layer is applied to execute a non-linear transformation. This operation enhances the representational capacity of the model, unifies feature dimensions across multiple scales, and yields the final fused feature maps. This provides a high-quality foundation for subsequent upsampling in U-VMamba and cross-modal integration in U-MTT. Following CAGF processing, non-pathological background details are effectively suppressed. This realizes a “global-to-local” guidance paradigm: the module preserves the fine-grained visual details extracted by U-VMamba while leveraging the global semantic information from U-MTT. Such a synergy significantly elevates the segmentation performance of the LViM model in complex orchard environments.

#### 3.2.5 Linguistic Cross-Nested (LCN)

The LCN module (Fig. 3(d)) is engineered to extract and transform disease-specific semantic attributes specifically tailored for apple leaf disease segmentation. A pre-trained BERT-base model (${BERT}_{12\_768\_12}$) is utilized to process critical pathological information—such as lesion color, morphology, and size—to generate semantic embeddings from expert-annotated labels. By converting these discrete semantic labels into 768-dimensional continuous word vectors, the LCN module establishes a foundational linguistic representation for cross-modal integration. This process is formally expressed in Equation (11):

(11) $$\begin{array}{c} X_{text}=BertEmbedding\left({text}_{i}\right) \end{array}$$

Where $X_{text}$ represents the standardized textual embeddings encoded by BERT, and ${text}_{i}$ denotes the descriptive text for the i-th apple disease category. These features complement the multi-granularity textual features outputs from AMBERT, providing domain-specific semantic priors that are highly relevant to apple disease scenarios for subsequent nested fusion.

The LCN module executes explicit tensor dimensionality transformations on both textual and visual features to facilitate efficient cross-modal fusion and ensure alignment between the two modalities. The fused textual tensors derived from AMBERT and BERT are reshaped into multi-scale representations with dimensions such as (2, 10, 28), (2, 10, 256), and (2, 10, 512), corresponding to the hierarchical stages of the U-VMamba downsampling path. Concurrently, the initial visual tensors from U-VMamba are processed to generate feature maps with dimensions of (2, 64, 224, 224), (2, 128, 112, 112), and (2, 256, 56, 56). This strategy establishes the necessary dimensionality context for cross-scale nested fusion, preserving fundamental pathological features while minimizing computational overhead. The LCN module, acting in synergy with the U-VMamba backbone, creates a nested fusion architecture that deeply integrates linguistic semantics and visual features across multiple scales. The process then proceeds to the fusion stage, where cross-modal interaction is first performed between the initial-layer visual tensors and the preceding textual features. This results in multi-dimensional fusion tensors of (2, 196, 64), (2, 196, 128), and (2, 196, 256), establishing an initial visual-semantic synergy as formally defined in Equation (12):

(12) $$\begin{array}{c} Y_{in,i}=VMamba\left(X_{img},X_{text}\right) \end{array}$$

where $Y_{in,i}$ represents the initial cross-modal fusion feature at the i layer. This feature facilitates the guidance of textual semantics toward visual representations by leveraging the cross-modal outputs from the MTT module. The LCN module is designed to implement a layer-by-layer, nested deep fusion mechanism based on this initial integration. The fusion output of each layer is downsampled and subsequently embedded into successive U-VMamba modules through a progressive process. By integrating the multi-scale global semantic features $X_{gcm}$, the module achieves robust cross-layer and cross-scale interaction. Specifically, the feature information from the preceding two nested fusions is combined to generate a multi-dimensional semantic nested vector, formally defined in Equation (13):

(13) $$\begin{array}{c} {\;Y}_{out,i}=\left[VMamba\left(Y_{in,i},X_{gcm}\right),VMamba\left(Y_{in,i-1},X_{gcm}\right),VMamba\left(Y_{in,i-2},X_{gcm}\right)\right] \end{array}$$

Where $Y_{in,i-1\;}$ and $Y_{in,i-2}$ are the cross-modal fusion features of the previous layer and the second previous layer, respectively, and $Y_{out,i}$ is the output feature of the nested fusion at the i layer. The model's semantic perception ability for apple leaf diseases is enhanced by the profound interaction between semantic-visual fusion features at multiple scales and levels, achieved through this layer-by-layer nested process.

Furthermore, the LCN module normalizes and scales the semantic nested tensors at each level to facilitate comprehensive multi-scale learning. Through a hybrid fusion strategy, it effectively couples the fine-grained visual characteristics of shallow lesions (e.g., textures and edges) with high-level semantic attributes (e.g., disease categories and overall distribution). As the central integration unit of the LViM model, the LCN module—in conjunction with the AMBERT encoder and MTT modules—constructs a collaborative, multi-layered cross-modal system. The resulting multi-scale nested features are subsequently fed into the CAGF module for further synthesis with global-local visual cues. By employing this cross-scale and cross-level nesting paradigm, the model establishes a semantic-guided visual encoding system. This synergy effectively mitigates the representational ambiguity inherent in unimodal visual encoding within complex field environments, thereby enhancing both feature perception and semantic segmentation precision for apple leaf diseases.

## 4 Experiments

To ensure the reproducibility of the LViM results and minimize experimental variability, all simulations were conducted within a unified hardware and software environment. The hardware infrastructure was provided by the AutoDL platform, while the software followed a standardized development environment to maintain experimental consistency. Comprehensive details regarding the hardware and software specifications are summarized in Table 1.

**Table 1:** The software and hardware environment of a computer.

Hardware Environment	CPU	AMD EPYC 7763
	RAM	64G
	GPU	NVIDIA RTX 4090
	Video Memory	24G
Software Environment	OS	Ubuntu 22.04
	CUDA Toolkit	12.2
	Python	3.8
	Pytorch-GPU	1.8.0

Following established protocols in existing research, all images in the multimodal apple leaf disease dataset were resized to a uniform resolution of 224×224 pixels. Data augmentation techniques—including adaptive brightness adjustment, random horizontal flipping, and random rotation—were implemented to enhance dataset diversity and mitigate overfitting. The model was trained using the Dice loss function with a batch size of 16. We employed the AdamW optimizer with an initial learning rate of 1e-4. A CosineAnnealingLR scheduler was utilized with a cycle of 100 epochs, spanning a total training duration of 200 epochs and a minimum learning rate of 1e-6 [39]. The VMamba visual encoder was initialized with pre-trained VMamba-S weights from ImageNet-1k, while the AMBERT text encoder also utilized its respective pre-trained weights. Parameters for all other modules were initialized following the Xavier uniform distribution [40]. The hardware environment featured an AMD EPYC 7763 CPU, 64 GB of RAM, and a single NVIDIA RTX 4090 GPU (24 GB VRAM). The software stack was built on Ubuntu 22.04, utilizing Python 3.8, PyTorch 1.8.0, and CUDA 12.2.

The performance of the proposed LViM framework was validated on our curated multimodal dataset across four evaluation metrics. Ablation studies were conducted to systematically analyze the architectural dependencies and justify the integration of each component. By comparing unimodal and multimodal cross-modal configurations, the specific advantages of our fusion framework were highlighted. Finally, the generalization capability of LViM was further evaluated on a public citrus disease dataset. Overall, these experiments demonstrate that the LViM framework achieves robust and superior segmentation performance across datasets of varying scales and complexities.

### 4.1 Evaluation criteria

To rigorously quantify the performance of the LViM framework, four widely recognized evaluation metrics were employed: Precision, Dice coefficient, mean Intersection over Union (mIoU), and Recall (Equation (14)–(17)). These metrics provide a comprehensive assessment of the model's segmentation fidelity and robustness. For the purpose of these calculations, the pixel-level classification outcomes are defined as follows:

1) True Positive (TP): Pathological pixels correctly identified as diseased by the model.
2) True Negative (TN): Healthy tissue pixels accurately classified as non-diseased (background).
3) False Positive (FP): Healthy pixels misclassified as pathological regions.
4) False Negative (FN): Pathological pixels that the model failed to detect.

Precision: Quantifies the percentage of predicted diseased areas that are truly diseased (TP). The formula is as follows:

(14) $$\begin{array}{c} \;Precision=\frac{TP}{TP+FP} \end{array}$$

Dice coefficient: A metric frequently used to assess model performance in image segmentation tasks, it measures the overlap between segmented predictions and true labels. It is defined as follows:

(15) $$Dice=\frac{2TP}{\left(TP+FN\right)+\left(TP+FP\right)}$$

mIoU: Indicates the extent of overlap between the annotated ground-truth regions and the predicted apple disease regions. The formula is as follows:

(16) $$\begin{array}{c} mIoU=\frac{1}{k+1}\sum_{i=0}^{k}\frac{\left|A_{i}\cap B_{i}\right|}{\left|A_{i}\cup B_{i}\right|} \end{array}$$

Where $A_{i}$ and $B_{i}$ denote the ground-truth region and the predicted region of the i-th class, respectively. The parameter k denotes the number of foreground disease categories, and k+1 represents the total number of classes involved in the mIoU calculation, including the background class. In this study, the foreground categories include Alternaria leaf spot, brown spot, gray spot, and rust.

Recall: Measures the proportion of actual positive samples that the model correctly predicts. The formula is as follows:

(17) $$Recall=\frac{TP}{TP+FN}$$

### 4.2 Ablation research

**Fig. 5:**
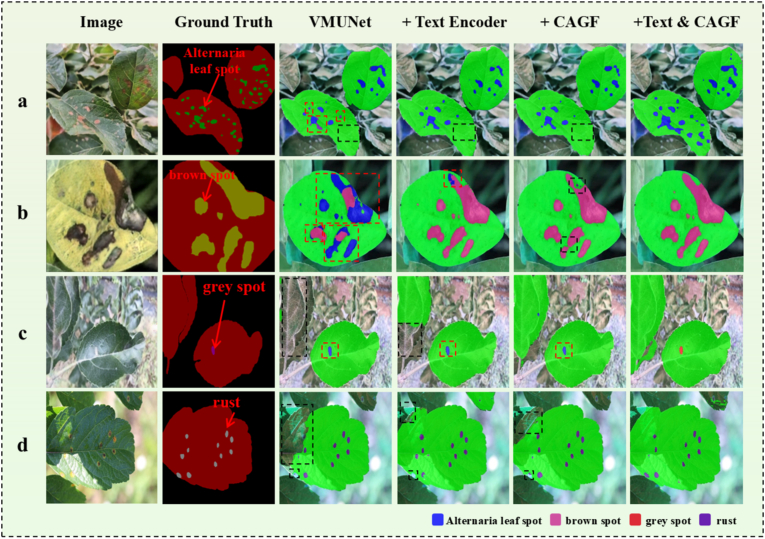
Illustrates the qualitative segmentation results of the ablation study. Black dashed boxes indicate regions of omitted detection (false negatives), while red dashed boxes signify areas of inter-class confusion or misclassification. For visualization purposes, the disease categories are color-coded as follows: Alternaria leaf spot (blue), brown spot (pink), gray spot (red), and rust (purple).

This study adopts VM-UNet as the baseline architecture. To evaluate the individual contributions and synergistic mechanisms of the core components within the LViM model, we conducted a series of ablation experiments on our custom-curated multimodal apple leaf disease dataset. By sequentially integrating the text encoders and Cross-Attention Gate Fusion (CAGF) modules, we systematically analyzed the impact of each component on model performance through both qualitative segmentation visualizations (Fig. 5) and quantitative metrics (Table 2).

**Table 2:** Presents a comparative analysis of various benchmark configurations evaluated on our in-house apple leaf disease dataset. It quantifies the segmentation performance—including Precision, Dice coefficient, mIoU, and Recall—as different modules are incrementally integrated. In addition, Parameters (M) reports the number of model parameters to evaluate the additional model complexity introduced by each module. The experimental progression demonstrates a cumulative improvement in performance, with the optimal results highlighted in bold.

Model	Precision (%)	Dice (%)	mIoU (%)	Recall (%)	Parameters (M)
VM-UNet(Baseline)	91.76	90.48	89.73	90.93	29.43
+ AMBERT-MTT branch	92.16	91.42	90.46	91.75	30.88
+ CAGF module	93.04	92.58	91.36	92.47	31.69
+ AMBERT-MTT branch & CAGF module	**95.81**	**94.73**	**94.22**	**95.16**	32.72

As a fundamental framework, the baseline VM-UNet exhibits basic disease segmentation capabilities. However, as illustrated in Fig. 5(a), (b), and (c), the model's ability to differentiate between visually similar disease categories—such as Alternaria leaf spot, brown spot, and gray spot—remains constrained. Substantial segmentation omissions were observed in the target leaf areas and across micro-scale lesion targets (Fig. 5(c) and (d)), indicating that the baseline model's diagnostic accuracy and classification reliability require further optimization.

To further analyze the model complexity introduced by the additional modules, the number of parameters is also reported in Table 2. The VM-UNet baseline contains 29.43M parameters. Compared with the baseline, introducing the AMBERT-MTT branch increases the parameter count by 1.45M, indicating that the Transformer-based multimodal branch introduces only a limited increase in model size while providing additional semantic modeling capability. When the CAGF module is independently incorporated, the parameter count increases by 2.26M compared with the baseline. Finally, the complete LViM model with both the AMBERT-MTT branch and the CAGF module increases the parameter count by 3.29M compared with the baseline. Although the proposed modules introduce additional parameters, the increase remains moderate relative to the performance gain, as the mIoU improves from 89.73% to 94.22%. This result indicates that LViM achieves a favorable balance between segmentation accuracy and parameter complexity.

Upon incorporating the text encoder into the baseline model, performance improved across all key metrics: Precision increased by 0.40%, Dice coefficient by 0.94%, mIoU by 0.73%, and Recall by 0.82%. This integration resulted in a consistent upward trend in overall performance. As shown in the visual results (Fig. 5(a) and (b)), the boundary delineations for Alternaria leaf spot and brown spot became more continuous and distinct, effectively mitigating the ambiguity between similar lesion types. Furthermore, previously undetected regions in Fig. 5(c) and (d) were partially identified. These findings demonstrate that the textual modality provides critical semantic priors, which enhance the model's ability to discriminate between similar pathological features and reduce misclassifications caused by complex background interference. However, the improvement brought by the text encoder alone is relatively limited because textual features mainly provide high-level category-related semantic information, such as lesion color, morphology, and disease-specific descriptions. These semantic priors help reduce inter-class ambiguity, but they do not directly impose pixel-level spatial constraints on lesion regions. Therefore, when the text encoder is used independently, it has limited ability to refine lesion boundaries or correct localization errors, which explains the relatively modest improvement in Precision.

The independent addition of the CAGF module to the VM-UNet yielded even more pronounced improvements: compared to the baseline, Precision rose by 1.28%, Dice coefficient by 2.10%, mIoU by 1.63%, and Recall by 1.54%. Visually, as shown in Fig. 5(b), brown spot regions are no longer misidentified as other diseases, and even the subtle, fine-grained rust lesions in Fig. 5(d) are effectively captured. The CAGF module strengthens the representation of key lesion features by calibrating visual attention and applying dynamic weighting, thereby reducing both false positives and omissions while significantly enhancing segmentation integrity and precision.

The LViM model achieves its peak performance when the text encoder and the CAGF module are jointly integrated. Compared to the baseline VM-UNet, all evaluation metrics demonstrate substantial gains: Precision increased by 4.05%, the Dice coefficient by 4.25%, mIoU by 4.49%, and Recall by 4.23%. Specifically, Fig. 5(a) reveals the successful segmentation of micro-scale Alternaria lesions; Fig. 5(b) showcases a more refined representation of the textural structures in brown spots; Fig. 5(c) demonstrates precise category identification for gray spots; and Fig. 5(d) yields segmentation masks that are highly congruent with the ground truth for rust.

These findings validate the synergy and functional complementarity between the text encoder and the CAGF module. While the text encoder serves to reduce cross-modal semantic ambiguity, the CAGF module focuses on the precise alignment and enhancement of visual features. Specifically, the text encoder provides disease-related semantic priors, enabling the model to better distinguish visually similar categories, whereas the CAGF module projects these semantic cues onto local visual representations through cross-attention gating. In this way, high-level textual semantics are converted into spatially guided attention weights, allowing the model to focus on lesion-relevant regions while suppressing background interference. Their simultaneous application significantly elevates segmentation performance in complex agricultural environments. The observed incremental trends in both quantitative metrics and visual comparisons confirm that the combination of these components provides greater benefits than a simple additive integration. This not only justifies the architectural design of LViM but also underscores the efficacy of cross-modal fusion for pathological segmentation. By deeply integrating linguistic semantic priors with visual features, the model effectively addresses persistent challenges, such as the detection of subtle lesions and the differentiation of visually similar pathologies. Consequently, this study provides a robust methodological framework for the intelligent monitoring and precise identification of apple leaf diseases in unstructured field settings.

### 4.3 State-of-the-art comparison

**Fig. 6:**
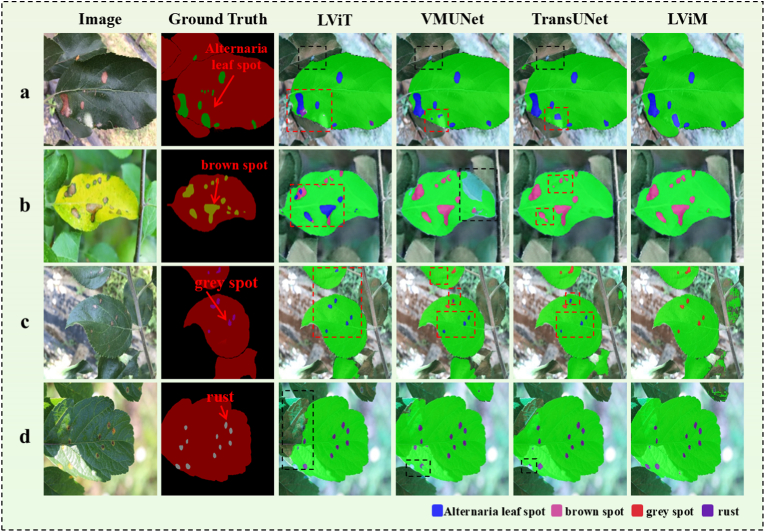
Qualitative comparison of segmentation results across various models. Black dashed boxes highlight omitted detections (false negatives), while red dashed boxes indicate regions of inter-class confusion or misclassification. The disease categories are color-coded as follows: Alternaria leaf spot (blue), brown spot (pink), gray spot (red), and rust (purple).

This study utilizes the custom-curated apple leaf disease dataset as a benchmark to objectively and systematically evaluate the performance advantages and practical utility of the LViM model. To provide a multi-faceted comparative analysis, seven prominent segmentation networks—UNet, DeepLabV3+, SegFormer [41], LViT [42], CTDUNet [43], VM-UNet, and TransUNet—were selected as reference models. Table 3 provides a comprehensive mIoU assessment alongside detailed segmentation metrics for each disease category. Furthermore, Fig. 6 presents a qualitative visual comparison of the segmentation results across all models, providing both quantitative and visual evidence to support the subsequent performance analysis.

**Table 3:** Comparative analysis of state-of-the-art methodologies on the apple leaf disease and pest dataset. The mIoU metric is employed to evaluate the segmentation performance across each disease category. Optimal results are highlighted in bold.

Model	Leaf	Alternaria spot	brown spot	gray spot	rust	mIoU (%)
UNet	91.43	70.53	71.49	78.32	85.60	79.47
DeeplabV3+	94.22	73.49	69.65	80.44	89.82	81.52
Segformer	**96.72**	76.52	75.34	84.58	90.41	84.71
LViT	92.67	69.44	72.47	86.21	88.76	81.91
CTDUNet	94.98	83.68	83.62	89.71	91.54	88.70
VM-UNet	94.46	85.12	85.33	90.47	93.28	89.73
TransUNet	95.57	88.23	89.07	91.84	96.85	92.31
LViM	96.49	**91.61**	**92.48**	**93.36**	**97.19**	**94.22**

Compared to the other baseline models, the conventional UNet architecture exhibits a relatively low overall mIoU. Its segmentation precision for brown spot and Alternaria leaf spot is inferior to that of most structurally enhanced advanced models. Qualitative analysis of the segmentation scenarios indicates that the vanilla encoder-decoder architecture of UNet struggles to adapt to complex field conditions. Specifically, when lesion textures closely resemble the natural leaf background or when significant environmental noise is present, UNet fails to extract fine-grained pathological features. This frequently results in segmentation artifacts and category misclassifications, highlighting the inherent limitations of traditional convolutional models in high-precision tasks.

Although DeeplabV3+, which integrates expansion convolution, improves the segmentation accuracy of rust, and the overall mIoU is slightly improved compared with UNet, the expansion of the feature acceptance field inevitably aggravates the loss of local detailed information. Therefore, its segmentation accuracy of brown spot is not as good as that of UNet, and it continues to face insurmountable challenges in identifying and segmenting small target lesions. In precise segmentation tasks, models based on hollow convolution often encounter this problem.

Among the Transformer-based models, SegFormer's hierarchical feature encoding structure demonstrates a significant advantage in segmenting the primary leaf regions, achieving the highest leaf-level accuracy among all comparative models. Consequently, its overall mIoU significantly outperforms that of traditional CNN-based frameworks. However, it is noteworthy that SegFormer exhibits substantial deficiencies in lesion-specific segmentation; its accuracy for Alternaria leaf spot and brown spot remains suboptimal compared to more sophisticated Transformer-based counterparts. This suggests that while SegFormer's feature extraction mechanism is effective at capturing large-scale global features, it lacks the representational capacity for micro-scale pathological details, thus failing to meet the rigorous requirements for precise disease identification. In contrast to SegFormer, the LViT model demonstrates a marked decrease in overall mIoU, with the most significant decline observed in Alternaria leaf spot segmentation. This indicates shortcomings in LViT's multi-scale feature fusion design, particularly its inability to appropriately allocate feature weights to account for disparate target scales. Consequently, it struggles to achieve a balanced feature representation between the overarching leaf structure and subtle lesions—a persistent challenge among existing unimodal Transformer-based segmentation models.

By refining feature fusion strategies, both CTDUNet and VM-UNet improve segmentation performance compared with earlier baseline models. Their overall mIoU is substantially higher than that of traditional architectures such as UNet, particularly in lesion-specific segmentation. However, compared with the proposed LViM model, these two models still show limited accuracy in distinguishing visually similar diseases, such as Alternaria leaf spot and brown spot. Fundamentally, these unimodal models struggle to resolve inter-class ambiguity using visual features alone, as they lack the capacity to incorporate cross-modal semantic information. TransUNet, which integrates Transformers into a UNet-based encoder-decoder structure, achieves the highest overall mIoU among all benchmarks except for LViM. Although its performance on rust is comparable to that of LViM, it performs less effectively in complex cases involving Alternaria leaf spot and brown spot. This is likely because its feature fusion is mainly limited to shallow concatenation, which fails to establish a deep coupling between visual features and linguistic semantic information, thereby limiting the benefits of full cross-modal integration.

A comprehensive comparison across all models underscores LViM's superior segmentation performance across all disease categories. Its performance metrics not only surpass advanced models like TransUNet but also demonstrate substantial gains over traditional CNNs, validating the rationality and efficacy of its architectural design. Notably, LViM significantly enhances the identification and segmentation accuracy of diverse lesion areas while maintaining global leaf segmentation precision comparable to SegFormer. This effectively addresses the inherent performance imbalance prevalent in traditional models, which often prioritize macroscopic leaf coverage at the expense of fine-grained spot characteristics—a key competitive advantage of the proposed method. Leveraging its multimodal enhancement mechanism, LViM better preserves marginal and internal details when encountering lesions with similar colors and complex textures. This effectively mitigates segmentation omissions and category confusion, demonstrating exceptional robustness and stability, particularly in high-precision tasks involving micro-scale lesions such as rust. Consequently, the LViM model is more adept at accurately segmenting apple leaf diseases and pests in complex agricultural environments. Compared to current state-of-the-art models, it more effectively meets the practical requirements of precision agricultural monitoring and provides significant technical insights for analogous tasks in crop disease classification.

### 4.4 Generalization experiment

**Fig. 7:**
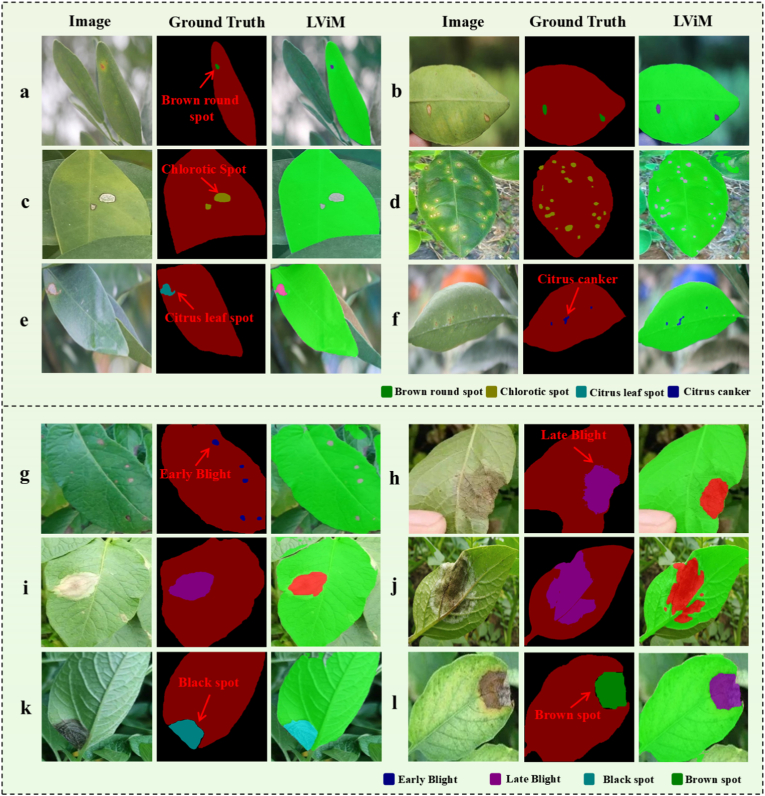
Qualitative segmentation results of LViM on citrus and potato leaf diseases in complex field environments. Panels (a–f) show citrus disease segmentation results, including brown round spot, chlorotic spot, citrus leaf spot, and citrus canker. Panels (g–l) show potato disease segmentation results, including early blight, late blight, black spot, and brown spot. Different disease categories are visualized using distinct colors.

Given the high morphological similarity of citrus and potato leaves to apple leaves, this study adopts a transfer learning paradigm to evaluate the LViM model's cross-species generalization. Specifically, the model weights pre-trained on the apple disease dataset were directly applied to citrus and potato disease detection without any task-specific fine-tuning for either crop. This approach rigorously assesses the parameter robustness of LViM in multi-crop scenarios. The experimental data included citrus and potato leaf disease samples, and Fig. 7 illustrates the model's segmentation performance under complex field conditions.

As shown in Fig. 7, LViM achieves effective segmentation of four typical citrus diseases without necessitating fine-tuning on citrus-specific data. For brown round spot (Fig. 7(a and b)), the model accurately localizes small, circular, and stellate lesions. The segmentation masks are highly congruent with the ground truth annotations, exhibiting sharp lesion boundaries and effectively distinguishing pathological tissues from healthy leaf areas. This demonstrates the model's cross-species capability in identifying distinct morphological features. For chlorotic spot (Fig. 7(c and d)), LViM precisely identifies chlorotic lesions characterized by grayish-white centers and brown margins, while successfully capturing the surrounding yellowing regions. The segmentation results comprehensively cover the necrotic centers without significant omissions or misclassifications, confirming the model's strong discriminative power for complex chlorotic symptoms. In the case of citrus leaf spot (Fig. 7(e)), the model successfully captures micro-scale lesions, with the segmentation boundaries closely adhering to the irregular morphology of the spots. This effectively minimizes confusion between the target lesions and the leaf background, highlighting its potential for fine-grained cross-species detection. Finally, for citrus canker (Fig. 7(f)), the model provides stable segmentation of characteristic oily, raised, and corky lesions. Even in scenarios with densely clustered or overlapping lesions, LViM clearly delineates individual boundaries, achieving high consistency with ground truth annotations and validating its generalizability to the unique features of bacterial diseases.

Furthermore, the potato disease results in Fig. 7(g–l) further demonstrate the cross-crop applicability of LViM. For early blight (Fig. 7(g)), the model successfully identifies scattered small lesions on the potato leaf surface, indicating its ability to detect relatively subtle disease spots. For late blight (Fig. 7(h–j)), LViM captures larger necrotic regions with irregular boundaries, showing stable segmentation performance for severe and spatially expanded lesions. For black spot (Fig. 7(k)), the model accurately localizes dark lesion areas with clear contrast against the leaf background. For brown spot (Fig. 7(l)), LViM effectively segments the main diseased region and maintains good consistency with the ground truth mask. These results suggest that the proposed semantic-visual fusion framework can generalize not only to citrus leaf diseases but also to potato leaf diseases with different leaf structures, lesion morphologies, and disease appearances.

Overall, within the cross-species transfer learning paradigm, the LViM model maintains robust segmentation performance across diverse citrus and potato disease categories and lesion scales, with no significant segmentation omissions or inter-class ambiguities observed. This demonstrates that the text-visual cross-modal alignment and lesion-specific attention mechanisms acquired during pre-training on apple diseases can be effectively transferred to different crop disease scenarios. This facilitates rapid adaptation without the necessity for species-specific retraining, providing an efficient and extensible methodological framework for the intelligent monitoring of multi-crop pests and diseases. These results preliminarily validate the feasibility of cross-species deployment and establish a solid foundation for extending the model to broader pathological identification tasks in other high-value specialty crops. In future work, we will further evaluate LViM on more diverse crops, disease categories, and field environments to comprehensively verify its generalization ability and practical robustness.

## 5 Conclusion

In complex orchard environments, apple leaf disease segmentation faces three primary obstacles: (1) high morphological similarity between disease regions and background textures, which complicates localization; (2) blurred or occluded lesions in overlapping leaves, which hinders complete feature capture; and (3) the limitations of unimodal models that rely solely on visual cues, leading to poor generalization on subtle pathological features. To address these challenges, we proposed LViM, a Mamba-Transformer dual-path U-Net framework that synergistically integrates textual and visual data.

By utilizing VMamba as the visual encoder, LViM effectively captures global context and long-range characteristic associations. Leveraging the unique advantages of SS2D and VSS blocks, the model enhances the representation of occluded lesions, thereby mitigating segmentation omissions in complex scenes. Concurrently, the textual modality bridges critical gaps in high-level semantic information, significantly improving the model's ability to distinguish lesions from complex backgrounds. We also introduced a multi-scale semantic cross-embedding network and a CAGF module, enabling the dynamic alignment of cross-modal features and facilitating hierarchical perception. This approach compensates for the inherent deficiencies of unimodal models by revealing latent semantic correlations between pathological signs and their environmental context.

Experimental results demonstrate that LViM achieves superior segmentation performance on apple leaf disease datasets. Compared to the baseline VM-UNet, our method yielded notable gains across all core evaluation metrics: Precision increased by 4.05%, Dice coefficient by 4.25%, mIoU by 4.49%, and Recall by 4.23%. Notably, LViM requires only 26% of the parameters of TransUNet, which achieved the highest mIoU among all other competing models. Furthermore, we developed a comprehensive multimodal dataset featuring four major disease categories across diverse natural environments, integrated with GPT-5-generated semantic descriptions to provide a robust foundation for future multimodal agricultural research. The proposed LViM framework is designed not only for apple leaf disease identification but also for pixel-level lesion localization in complex orchard environments. Compared with classification-based methods, semantic segmentation can provide lesion masks with explicit spatial boundaries, which makes it possible to estimate lesion area and further support disease severity grading. Therefore, the proposed method is more suitable for continuous disease monitoring and precision disease management in practical agricultural scenarios.

For field deployment, the proposed LViM framework does not require users to manually provide textual descriptions for each input image. In practical orchard applications, users only need to capture leaf images, while the disease-related textual prompts can be predefined according to disease categories or automatically retrieved from a built-in disease-description library. These textual prompts serve as auxiliary semantic priors to support semantic-visual feature fusion without increasing the operational burden on users. Meanwhile, certain limitations remain. While LViM performs well under standard conditions, its precision in extreme environmental settings, such as severe overexposure or ultra-low light, can be further optimized. In addition, disease symptoms may vary across different infection stages. Early-stage lesions are usually small and low-contrast, whereas severe infections may involve lesion adhesion and blurred boundaries. Therefore, future work will further evaluate the performance of LViM under different disease development stages to better support continuous disease monitoring.

## Availability of supporting data

The original PlantVillage dataset is available at https://github.com/spMohanty/PlantVillage-Dataset. The curated multimodal apple leaf disease dataset constructed in this study has been publicly released at https://github.com/csuft1906ll/LViM, and the code and models developed in this study will be released in the same repository upon acceptance of the paper.

## CRediT authorship contribution statement

Jiale Chen: Writing – original draft, Formal analysis, Methodology, Data curation, Conceptualization. Wei Shi: Data curation, Investigation, Visualization, Conceptualization. Ziyang Shi: Formal analysis, Validation, Investigation. Lin Li: Writing – review & editing, Formal analysis, Funding acquisition. Hao Zhou: Data curation. Xuanhao Li: Data curation.

## Funding

This work was supported by the 10.13039/501100001809National Natural Science Foundation of China (Grant No. 61902436); the Education Department Key Program of Hunan Province (Grant No. 21A0160); and the Hunan Provincial Science and Technology Innovation Program Project (Grant No. YLS-2025- ZY02048).

## Declaration of competing interest

The authors declare that they have no known competing financial interests or personal relationships that could have appeared to influence the work reported in this paper.

## Data Availability

The data supporting the findings of this study are available from the corresponding author upon reasonable request.
